# Epigenetic and gene expression changes of neuronal cells from MSA patients are pronounced in enzymes for cell metabolism and calcium-regulated protein kinases

**DOI:** 10.1007/s00401-021-02357-5

**Published:** 2021-08-09

**Authors:** Laura de Boni, Gilles Gasparoni, Anna Welle, Sascha Tierling, Ina Schmitt, Jörn Walter, Jochen Walter, Ullrich Wüllner

**Affiliations:** 1 Department of Neurology, University Hospital Bonn, Venusberg-Campus 1, Bonn, Germany; 2 Department of Genetics/Epigenetics, Saarland University, Campus Saarbrücken, Building A2 4, Saarbrücken, Germany

In 2020, Bettencourt et al. [1] investigated total DNA methylation alterations in white matter from patients with Multiple System Atrophy (MSA). They identified *HIP1*, *LMAN2* and *MOBP* amongst the most differentially methylated loci. However, MSA is not only characterised by abnormal Glial Cytoplasmic Inclusions (GCIs) but also Neuronal Cytoplasmic Inclusions (NCIs) containing the α-synuclein (αSYN) protein [3, 4]. Despite the extensive neuropathological characterisation and the identification of genetic risk loci, the pathogenesis of MSA remains largely unknown [2]. Novel genetic or epigenetic clues of white and grey matter gene regulation in MSA patients linked to the pathophysiology are, therefore, urgently needed for a better understanding of the pathogenesis.

To address this issue and in addition to the aforementioned study, we carried out a total DNA methylation analysis (EPIC array, Illumina) covering 850 K CpGs in the genome of FACS-sorted NeuN-positive neuronal nuclei from the occipital cortex (OC) of MSA patients and controls (Material and Methods, online resource; Table 1, online resource). We chose this low affected brain region in MSA patients to detect presumed pre-existing, possible disease-causing epigenetic changes leaving out epigenetic changes due to the disease itself.

Most of the top 10 K ranking CpGs (*n* = 9638) using a combined rank analysis (see online resource for material and methods) were hypermethylated in MSA patients compared to controls (Fig. 1a, b). The majority of those hypermethylated CpGs was located at gene bodies (*n* = 3440, 36%), intergenic regions (not annotated, *n* = 2601, 27%) and upstream of transcription start site (TSS) 1500 (*n* = 1725, 18%, Fig. 1c). Hypomethylated CpGs in MSA patients were mainly located in gene bodies (*n* = 108, 30%, Fig. 1c). GO analysis based on these top 10 K ranking CpGs identified several CpGs enriched in the biological processes and molecular function of guanosinetriphosphatase activity and adenosinetriphosphatase coupled to transmembrane transporter activity (Fig. 1d). Cellular components showed an enrichment of the septin protein family and pathways of calcium signalling (Fig. 1d). As not all CpGs of a respective gene are represented on the EPIC array, we evaluated which genes in the top 10 k ranking CpGs are significantly enriched in the dataset according to their number of CpGs on the array and the overall number of CpGs on the gene. Thus, we identified the enriched significant genes in the top 10 k data set according to the combined rank value. The majority of these genes in the top 20 were associated with inflammatory/immune responses and transcriptional regulation (*n* = 7, Table 2, online resource). Interestingly, all CpGs of the top 20 genes were hypermethylated in MSA patients independently of their location on the gene (Table 2, online resource).

**Fig. 1:**
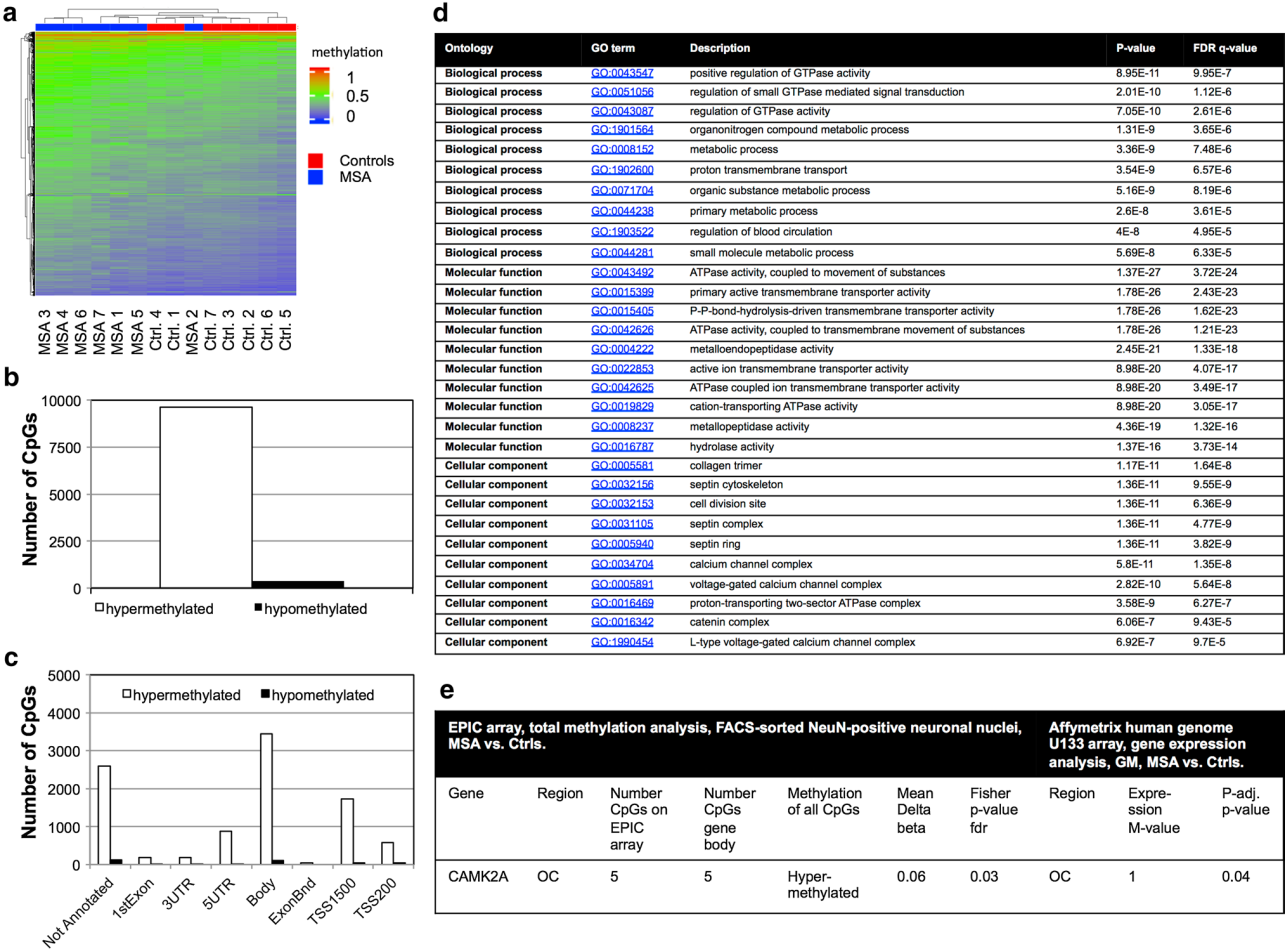
DNA methylation and gene expression analysis in FACS-sorted neurons from MSA patients and healthy controls. **a** Methylation heatmap for the top 10 K ranking CpGs according to the combined rank metrics from RnBeads. **b** Number of hypermethylated and hypomethylated CpGs from the top 10 K CpGs in MSA patients compared to controls.** c** Distribution of the top 10 K CpGs at genomic annotations. *Ctrl.* control, *ExonBnd* exon boundaries, *TSS* transcription start site, *UTR* untranslated region. **d** Top 10 GO enriched terms of each ontology group of the top 10 K ranked CpGs from the DNA methylation analysis of NeuN-positive neuronal nuclei. **e** Overlap of CpGs and DEGs according to the number of CpGs present on the EPIC array of a respective gene corrected for the overall number of CpGs on the gene. *Ctrls.* controls, *p-adj. p-value* *p*-adjusted *p*-value, *OC* occipital cortex, *GM* grey matter, *5mC* 5-methylcytosine, *fdr* false discovery rate. Data analysis is based on a Bayesian model calculation of the m-value. M + 1 = increased expression, twofold change of mean expression

Comparing our array-based top 10 K ranked neuronal CpGs to array-based transcriptomic data from MSA patients and controls (Affymetrix human genome U133, Thermo Fisher Scientific, Table 3, online resource), we identified 34 genes with overlapping CpGs and differentially expressed genes (DEG) in the grey matter (GM) of the OC (Table 4, online resource). All CpGs were hypermethylated in MSA patients and 28 of 34 corresponding genes were downregulated (Table 4, online resource). GO analysis of our top 10 K CpG ranked dataset showed an overlap of hypermethylated CpGs and upregulated DEGs associated with voltage-gated calcium channel complexes (*p* = 2.6E-6) and calcium channel complexes (*p* = 2.4E-5). According to the number of CpGs linked to a specific gene and the number of CpGs represented on the EPIC array, only one overlapping hypermethylated CpG/upregulated DEG pair (located at the gene body), *CAMK2A,* could be identified (Fig. 1e).

When comparing the top 10 K CpGs of the present study to the study of Bettencourt et al. [1] who analysed total methylation in white matter (WM) tissue of MSA patients (Table 5, online resource), only three overlapping genes (Table 6, online resource) were identified. In contrast, Rydbirk et al. studied 5-methylcytosine and 5-hydroxymethylcytosine separately in bulk brain tissue from MSA patients (Table 5, online resource) [7] and 17 CpGs located on 12 genes overlapped with our 10 K CpGs (Table 7, online resource). However, *AREL1*, the main locus identified in the study of Rydbirk et al. was not present in our top 10 K dataset of neuronal preparations. Overall, these comparisons demonstrate the cell-type-specific DNA methylation differences in MSA patients and the necessity to analyse tissue-specific preparations.

We also evaluated two other MSA transcriptomic studies (Table 5, online resource) [5, 6]: Piras et al. investigated WM of the cerebellum (CE) and laser-microdissected (LCM) oligodendrocytes. Mills et al. focussed on DEGs upregulated in WM vs. GM and GM vs. WM of MSA patients. Comparing our DEG dataset with DEGs from the study of Piras et al., only one overlapping DEG, *Notch2*, which was downregulated twofold in GM (p adj. p-value 0.03) and upregulated in WM (log2-fold 0.7, p adj. p-value 0.04) was identified. Comparing our own GM DEGs from the occipital cortex to GM DEGs from frontal cortex of the study from Mills et al., we only identified seven overlapping DEGs including *CAMKK2* which was differentially regulated in the brain regions (Table 8, online resource).

To summarise, our study, although performed with a small sample size, clearly demonstrates distinct DNA methylation and gene expression changes in neurons from MSA patients. Our comparative analyses of recently published data, albeit with different study designs and statistical analysis on DNA methylation and transcriptomics in MSA patients, showed that MSA patients not only exhibit specific differences in epigenetic and gene expression regulation compared to controls, but also between neuronal and glial cells. Thus, it will be necessary shed further light on epigenetic and gene expression analysis in post-mortem brain tissue comparing grey and white matter alterations between different MSA subtypes in different brain regions.

## Supplementary Information

Below is the link to the electronic supplementary material.

Supplementary file1 (DOCX 82 KB)
