# Supplementary material for: Epigenetic and gene expression changes of neuronal cells from MSA patients are pronounced in enzymes for cell metabolism and calcium-regulated protein kinases
Source: Acta Neuropathol. 2021 Aug 9;142(4):781–3. doi: 10.1007/s00401-021-02357-5 (PMC8423633; doi:10.1007/s00401-021-02357-5)
Supplement: Supplementary file 1 — Supplementary file1 (DOCX 82 KB) [file 401_2021_2357_MOESM1_ESM.docx]

Epigenetic and gene expression changes of neuronal cells from MSA patients are pronounced in enzymes for cell metabolism and calcium-regulated protein kinases.

Laura de Boni^1^, Gilles Gasparoni^2^, Anna Welle^2^, Sascha Tierling^2^, Ina Schmitt^1^, Jörn Walter^2^, Jochen Walter^1^, Ullrich Wüllner^1^

^1^ Department of Neurology, University Hospital Bonn, Venusberg-Campus 1, Bonn, Germany

^2^ Department of Genetics/Epigenetics, Saarland University, Campus Saarbrücken, Building A2 4, Saarbrücken, Germany.

Correspondence to: [laura.deboni@ukbonn.de](mailto:laura.deboni@ukbonn.de)

**Material and Methods**

**Human post-mortem brain material**

Brain tissue was obtained from the Queen Square Brain Bank in London and the Neurobiobank at the Center for Neuropathology in Munich. All samples were used with the consent of the local ethics committee.

**Isolation of neuronal nuclei**

Isolation of neuronal nuclei was performed according to the protocol published in Welle et al. 2021 [5]. Briefly, neuronal nuclei were extracted from frozen occipital cortex of MSA patients and controls. The tissue was manually homogenized and the nuclei suspension filtered through a 150 μm strainer followed by a 20 μm strainer (CellTrics, Symex Partec) to remove non-dissociated tissue. Further cell debris was removed by a centrifugation step in 25 % Percoll solution (Sigma Aldrich) for 15 min at 4 °C. Nuclei were washed, supplemented with 1x cOmplete, EDTA-free Protease Inhibitor Cocktail and kept in blocking

solution (3 % bovine serum albumin, 2 % normal goat serum in 1x PBS; pH 7.4) for 30 min at 4 °C. Anti-NeuN-Alexa488 antibody (MAB377X; Merck) was diluted 1:1,000 in the same volume of blocking solution as the nuclei. Nuclei and antibodies were incubated for 1 hour in the dark at 4 °C. The antbody solution was removed, the nuclei resuspended in blocking solution supplemented with 1 μg/ml prior to FACS analysis. The amount of isolated nuclei varied due to the amount of neuronal cells present in the tissue. The amount of neuronal nuclei ranged from 426,609.00 to 2,412,640.00.

**FACS analysis**

FACS analysis was carried out using a BD FACSaria III. Sorting gates were set based on PI and Alexa488 signal intensities. Nuclei pellets were stored at -80 °C.

**DNA extraction**

Nuclei pellets were digested with Proteinase K and DNA was extracted using Phenol-Chloroform/Isoamyl alcohol and ice cold ethanol. The pelleted DNA was dried and solubilized in 1x TE buffer. DNA amounts were assessed using a Qubit fluorometer according to the manufacturer’s instructions.

**RNA extraction**

RNA extraction was performed using cortex tissue and the RNeasy Mini-Kit (Qiagen) according to the manufacturer’s instructions.

**Bisulfite conversion**

Bisulfite conversion was performed using the Zymo EZ DNA Methylation-Gold Kit (Zymo Research) according to the manufacturer’s instructions. The input DNA for each bisulfite conversion reaction was 500 ng.

**EPIC array DNA methylation analysis**

The Infinium MethylationEPIC Array was used to analyse genome-wide total DNA methylation alterations. The analysis was performed according to the manufacturer’s instructions. Total methylation levels of over 850,000 methylations sites throughout the human genome at single nucleotide resolution were assessed.

**Affymetrix GeneChip Human Genome U133 analysis**

The Affymetrix GeneChip Human Genome U133 was used to carry out transcriptomic analysis. The analysis was performed according to the manufacturer’s instructions.

**Statistical analysis**

DNA methylation: DNA methylation has been analyzed using the RnBeads package[1, 4]. RnBeads is an R package (<https://www.r-project.org/>) [4] for comprehensive analysis of DNA methylation data obtained with any experimental protocol that provides single-CpG resolution. We used a combined rank analysis of methylated CpGs considering p-value rating, bead coverage and effect sizes. Single nucleotide polymorphisms located on CpGs were excluded from the analysis. RnBeads generates quality control plots both for Infinium EPIC array and bisulfite sequencing data. The quality control is implemented in the pipeline (Sample-independent controls: e.g. hybridization, target removal; Sample-dependent controls: e.g. bisulfite conversion, negative control, normalization). We report nominal p-values and the combined rank metrics as calculated by RnBeads[4]. Gene enrichement analysis: The analysis corrects for the number of analysed CpGs of their respective gene identifying the CpGs and thus the enriched significant genes in the top 10k data set according to the combined rank value.

Transcriptomics: For the expression analysis, a factorial design taking into account different features of the samples was applied and a paired analysis performed. Depending on the factorial design, a linear model was created describing the coefficients of the expression profile of a certain gene. An M value was calculated for the pairwise comparisons. The M values are equivalent to the log2 fold change. E.g. an M value of 1 is equivalent to a double increase in gene expression. For all gene transcripts, a resulting p-value was calculated and corrected for multiple testing according to Benjamini-Hochberg (p<0.05).

Gene ontology (GO) analysis: For the GO enrichement analysis, we used the Gene Ontology enRIchment anaLysis and visuaLizAtion tool (GOrilla)[2, 3]. All top 10k ranked CpGs based on the combined rank were used for the GO analysis. We used homo sapiens as the selected organism and a single ranked list of genes as running mode.

1. Assenov Y, Müller F, Lutsik P, Walter J, Lengauer T, Bock C (2014) Comprehensive analysis of DNA methylation data with RnBeads. Nat Methods 11:1138–40. doi: 10.1038/nmeth.3115

2. Eden E, Lipson D, Yogev S, Yakhini Z (2007) Discovering motifs in ranked lists of DNA sequences. PLoS Comput Biol 3:0508–0522. doi: 10.1371/journal.pcbi.0030039

3. Eden E, Navon R, Steinfeld I, Lipson D, Yakhini Z (2009) GOrilla: a tool for discovery and visualization of enriched GO terms in ranked gene lists. BMC Bioinformatics 10:48. doi: 10.1186/1471-2105-10-48

4. Müller F, Scherer M, Assenov Y, Lutsik P, Walter J, Lengauer T, Bock C (2019) RnBeads 2.0: comprehensive analysis of DNA methylation data. Genome Biol 20:55. doi: 10.1186/s13059-019-1664-9

5. Welle A, Kasakow C V., Jungmann AM, Gobbo D, Stopper L, Nordström K, Salhab A, Gasparoni G, Scheller A, Kirchhoff F, Walter J (2021) Epigenetic control of region-specific transcriptional programs in mouse cerebellar and cortical astrocytes. Glia 1–18. doi: 10.1002/glia.24016

Table 1 DNA methylation analysis of NeuN-positive neuronal nuclei. Overview of individuals.

| Individual | Brain region | Gender | Age at death | PMI [hrs.] | pH | max. L-Dopa [mg/d] | Disease duration [yrs.] | MSA subtype | GCIs or NCIs OC | Concomitant pathology |
| --- | --- | --- | --- | --- | --- | --- | --- | --- | --- | --- |
| MSA 1 | occipital cortex | male | 65 | n.a. | n.a. | n.a. | n.a. | n.a. | n.a. | tangles |
| MSA 2 | occipital cortex | male | 70 | 24 | n.a. | 700 | 8 | OPCA + SND | n.a. | Occasional tangles |
| MSA 3 | occipital cortex | female | 70 | 25 | 6.56 | 0 | 5 | OPCA + SND | n.a. | Occasional tangles |
| MSA 4 | occipital cortex | female | 75 | 30 | 6.3 | 1000 | 9 | Predominant SND | n.a. | Occasional tangles |
| MSA 5 | occipital cortex | female | 65 | 28 | 6.5 | 1000 | 7 | Predominant SND | GCIs | Occasional tangles |
| MSA 6 | occipital cortex | male | 61 | 11 | 6.6 | 1600 | 5 | Predominant SND | n.a. | none |
| MSA 7 | occipital cortex | male | 72 | 36 | 6.43 | n.a. | n.a. | OPCA + SND | GCIs | none |
| Control 1 | occipital cortex | female | 74 | 17 | n.a. | n.a. | n.a. | n.a. | n.a. | Occasional tangles |
| Control 2 | occipital cortex | female | 83 | 20 | 6.6 | n.a. | n.a. | n.a. | n.a. | none |
| Control 3 | occipital cortex | female | 84 | 82 | 6.3 | n.a. | n.a. | n.a. | n.a. | Occasional tangles/plaques |
| Control 4 | occipital cortex | female | 69 | 38 | n.a. | n.a. | n.a. | n.a. | n.a. | none |
| Control 5 | occipital cortex | male | 73 | n.a. | n.a. | n.a. | n.a. | n.a. | n.a. | none |
| Control 6 | occipital cortex | male | 79 | 56 | 6.6 | n.a. | n.a. | n.a. | n.a. | none |
| Control 7 | occipital cortex | male | 86 | 53 | 6.7 | n.a. | n.a. | n.a. | n.a. | none |

OC = Occipital cortex, PMI = post mortem interval, hrs. = hours, yrs. = years, SND = striatonigral degeneration (MSA-P), OPCA = olivopontocerebellar atrophy (MSA-C), GCI = glial cytoplasmic inclusion, NCIs = neuronal cytoplasmic inclusions

Table 2 Top 20 significant genes in the top 10k dataset according to their number of CpGs on the array and the overall number of CpGs on the gene.

| Gene | Gene Name | Function | Total No. CpGs on EPIC  array | No. CpGs gene body | No. CpGs 5'UTR | No.  CpGs TSS  200 | No. CpGs TSS  1500 | No.  CpGs  1st Exon | No.  Hyper-methy-  lated  CpGs MSA vs. Ctrl. | No. Hypo-methy  lated CpGs MSA vs. Ctrl. | Mean delta beta of all CpGs MSA vs. Ctrl. | Fisher  p-value  fdr |
| --- | --- | --- | --- | --- | --- | --- | --- | --- | --- | --- | --- | --- |
| NCOR2 | Nuclear receptor corepressor 2 | transcriptional corepressor | 29 | 27 | 2 |  |  |  | 29 | 0 | 0.07 | 1.60E-13 |
| GNMT | Glycine N-methyltransferase | catalyzes the methylation of glycine | 14 | 1 |  | 5 | 7 | 1 | 14 | 0 | 0.08 | 6.43E-13 |
| NFIX | Nuclear factor 1 X-type | transcription and replication | 16 | 14 |  |  | 2 |  | 16 | 0 | 0.07 | 2.84E-09 |
| HCG4P6 | HLA Complex Group 4B | immune response | 11 |  |  | 1 | 10 |  | 11 | 0 | 0.11 | 2.84E-09 |
| AQP4 | Aquaporin-4 | brain water homeostasis | 9 | 4 |  | 2 | 3 |  | 9 | 0 | 0.08 | 3.23E-09 |
| IFIH1 | Interferon-induced helicase C domain-containing protein 1 | innate immune receptor | 9 |  | 1 | 4 | 4 |  | 9 | 0 | 0.06 | 1.96E-08 |
| KLK7 | Kallikrein-7 | cleavage of proteins, inflammation | 9 |  | 2 | 4 | 3 |  | 9 | 0 | 0.09 | 2.38E-08 |
| RPSAP58 | Ribosomal Protein SA Pseudogene 58 | pseudogene | 7 | 3 |  | 4 |  |  | 7 | 0 | 0.06 | 2.04E-06 |
| MGST2 | Microsomal glutathione S-transferase 2 | catalyzes several different glutathione-dependent reactions | 8 | 1 | 1 |  | 1 | 5 | 8 | 0 | 0.07 | 6.32E-06 |
| HCG9 | HLA Complex Group 9 | immune response | 9 | 9 |  |  |  |  | 9 | 0 | 0.09 | 9.28E-06 |
| NR3C1 | Glucocorticoid receptor | transcription factor, modulation of TFs | 11 | 4 | 7 |  |  |  | 11 | 0 | 0.08 | 9.50E-06 |
| UNC5A | Netrin receptor UNC5A | receptor for netrin required for axon guidance | 10 | 10 |  |  |  |  | 10 | 0 | 0.07 | 1.23E-05 |
| ZADH2 | Prostaglandin reductase 3 | functions as 15-oxo-prostaglandin 13-reductase | 7 | 7 |  |  |  |  | 7 | 0 | 0.07 | 1.38E-05 |
| SORL1 | Sortilin-related receptor | sorting receptor that directs several proteins | 11 | 10 |  |  | 1 |  | 11 | 0 | 0.07 | 2.03E-05 |
| CABP1 | Calcium-binding protein 1 | modulates calcium-dependent activity of inositol 1,4,5-triphosphate receptors | 8 | 5 | 1 |  | 2 |  | 8 | 0 | 0.08 | 3.47E-05 |
| ZFYVE21 | Zinc finger FYVE domain-containing protein 21 | cell adhesion | 8 | 8 |  |  |  |  | 8 | 0 | 0.08 | 5.98E-05 |
| NLRP2 | NLR Family Pyrin Domain Containing 2 | inflammation/immune responses | 7 |  | 2 | 3 | 2 |  | 7 | 0 | 0.11 | 9.71E-05 |
| IL17RC | Interleukin-17 receptor C | inflammation/immune responses | 6 | 1 | 1 | 2 | 2 |  | 6 | 0 | 0.07 | 9.71E-05 |
| PDYN | Proenkephalin-B | opiod activity, responses to stress | 6 |  |  | 1 | 4 | 1 | 6 | 0 | 0.06 | 9.71E-05 |
| NIPAL4 | Magnesium transporter NIPA4 | Mg^2+^ transporter | 6 |  |  | 1 | 5 |  | 6 | 0 | 0.05 | 1.17E-04 |

No. = number, Ctrl. = Control

Table 3 Transcriptomic studies. Overview of individuals.

| Individual | Brain region | Gender | Age at death (yrs.) | PMI [hrs.] | pH | max. L-Dopa [mg/d] | | Disease duration [yrs.] | MSA subtype | GCIs or NCIs  OC | Concomitant pathology |
| --- | --- | --- | --- | --- | --- | --- | --- | --- | --- | --- | --- |
| MSA 1 | occipital cortex | male | 72 | 36 | 6.4 | n.a. | | n.a. | OPCA and SND | GCIs | none |
| MSA 2 | occipital cortex | male | 61 | 11 | 6.6 | 1600 | 5 | | Predominant SND | n.a. | none |
| MSA 3 | occipital cortex | male | 78 | 23 | 6.4 | 1200 | 6 | | Predominant SND | n.a. | Occasional tangles |
| MSA 4 | occipital cortex | female | 65 | 28 | 6.5 | 1000 | 7 | | Predominant SND | GCIs | Occasional tangles |
| Control 1 | occipital cortex | male | 86 | 53 | 6.7 | n.a. | n.a. | | n.a. | n.a. | none |
| Control 2 | occipital cortex | female | 81 | 14 | 6.4 | n.a. | n.a. | | n.a. | n.a. | Occasional tangles |
| Control 3 | occipital cortex | male | 86 | 23 | 6.6 | n.a. | n.a. | | n.a. | n.a. | none |
| Control 4 | occipital cortex | male | 85 | 43 | 6.7 | n.a. | n.a. | | n.a. | n.a. | none |

OC = Occipital cortex, Yrs. = years, PMI = post mortem interval, hrs. = hours, SND = striatonigral degeneration (MSA-P), OPCA = olivopontocerebellar atrophy (MSA-C), GCI = glial cytoplasmic inclusion, NCIs = neuronal cytoplasmic inclusions

Table 4 Array-based analysis and overlap of differentially methylated CpGs (top 10k ranked CpGs, EPIC array) and expressed genes (Affymetrix array) carried out for the present study

|  |  |  |  | EPIC array, total methylation analysis,  FACS-sorted neurons, MSA vs. Ctrls. | | | Affymetrix gene expression analysis,  GM, MSA vs. Ctrls. | |
| --- | --- | --- | --- | --- | --- | --- | --- | --- |
| Gene name | CpG ID | Chromosome | Gene location | Combined rank | Delta beta MSA vs. Ctrls. | Methylation  difference  MSA vs. Ctrls. | P adj.P.Val | Occipital Cortex |
| ACOT2 | cg25598083 | chr14 | TSS1500 | 31807 | 0.09 | hypermethylated | 0.0068 | -1 |
| AGXT2L1 | cg07890104 | chr4 | TSS1500 | 44975 | 0.05 | hypermethylated | 0.0062 | -1 |
| ATP1A2 | cg18454248 | chr1 | Body | 24476 | 0.06 | hypermethylated | 0.0063 | -1 |
| ATP1A2 | cg03525467 | chr1 | Body | 47321 | 0.05 | hypermethylated | 0.0063 | -1 |
| ATXN1 | cg24067911 | chr6 | 5UTR | 17205 | 0.07 | hypermethylated | 0.0002 | 1 |
| ATXN1 | cg26309538 | chr6 | 5UTR | 26603 | 0.08 | hypermethylated | 0.0002 | 1 |
| ATXN1 | cg27395391 | chr6 | 5UTR | 39234 | 0.08 | hypermethylated | 0.0002 | 1 |
| BMP7 | cg03573446 | chr20 | TSS200 | 32855 | 0.04 | hypermethylated | 0.0419 | -1 |
| CAMK2A | cg00261592 | chr5 | Body | 11987 | 0.07 | hypermethylated | 0.0404 | 1 |
| CAMK2A | cg07622079 | chr5 | Body | 22849 | 0.06 | hypermethylated | 0.0404 | 1 |
| CAMK2A | cg19513232 | chr5 | Body | 23038 | 0.07 | hypermethylated | 0.0404 | 1 |
| CAMK2A | cg22642062 | chr5 | Body | 29696 | 0.06 | hypermethylated | 0.0404 | 1 |
| CAMK2A | cg04025535 | chr5 | Body | 33450 | 0.06 | hypermethylated | 0.0404 | 1 |
| CAMKK2 | cg03391567 | chr12 | 5UTR | 22351 | 0.07 | hypermethylated | 0.0033 | -1 |
| CAMKK2 | cg13374726 | chr12 | 1stExon | 47833 | 0.04 | hypermethylated | 0.0033 | -1 |
| COL16A1 | cg25621182 | chr1 | Body | 26266 | 0.07 | hypermethylated | 0.0110 | -1 |
| COL5A2 | cg24900931 | chr2 | Body | 43660 | 0.06 | hypermethylated | 0.0000 | -1 |
| CTBP1 | cg08948841 | chr4 | TSS1500 | 9886 | 0.19 | hypermethylated | 0.0129 | 1 |
| CTBP1 | cg25897951 | chr4 | TSS1500 | 24849 | 0.16 | hypermethylated | 0.0129 | 1 |
| CTBP1 | cg16399632 | chr4 | TSS1500 | 28716 | 0.22 | hypermethylated | 0.0129 | 1 |
| CTBP1 | cg15586393 | chr4 | TSS1500 | 43383 | 0.13 | hypermethylated | 0.0129 | 1 |
| CTSH | cg02519044 | chr15 | Body | 23183 | 0.06 | hypermethylated | 0.0151 | -1 |
| EDNRB | cg21675115 | chr13 | TSS1500 | 30829 | 0.05 | hypermethylated | 0.0358 | -1 |
| EFEMP1 | cg20786074 | chr2 | 1stExon | 28724 | 0.05 | hypermethylated | 0.0206 | -1 |
| EFEMP1 | cg05385513 | chr2 | TSS200 | 38874 | 0.04 | hypermethylated | 0.0206 | -1 |
| ELP4 | cg17696468 | chr11 | Body | 6395 | 0.09 | hypermethylated | 0.0383 | -1 |
| FERMT2 | cg15646987 | chr14 | TSS1500 | 25537 | 0.05 | hypermethylated | 0.0170 | -1 |
| FERMT2 | cg01820754 | chr14 | TSS1500 | 39170 | 0.04 | hypermethylated | 0.0170 | -1 |
| GPX3 | cg17820459 | chr5 | Body | 4521 | 0.07 | hypermethylated | 0.0062 | 1 |
| GPX3 | cg01378878 | chr5 | TSS200 | 10773 | 0.06 | hypermethylated | 0.0062 | 1 |
| MAP9 | cg13347038 | chr4 | TSS1500 | 32452 | 0.06 | hypermethylated | 0.0025 | -1 |
| MYLK | cg23184556 | chr3 | 5UTR | 31519 | 0.08 | hypermethylated | 0.0100 | -1 |
| MYO1B | cg11508595 | chr2 | 5UTR | 33919 | 0.08 | hypermethylated | 0.0227 | 1 |
| NTRK2 | cg22402007 | chr9 | TSS1500 | 48142 | 0.04 | hypermethylated | 0.0002 | -1 |
| PDLIM5 | cg22100409 | chr4 | 5UTR | 8825 | 0.07 | hypermethylated | 0.0443 | -1 |
| PICK1 | cg12821663 | chr22 | Body | 36850 | 0.06 | hypermethylated | 0.0448 | 1 |
| PMP22 | cg05738748 | chr17 | TSS1500 | 31075 | 0.09 | hypermethylated | 0.0408 | -1 |
| PPAP2B | cg11701090 | chr1 | Body | 13305 | 0.06 | hypermethylated | 0.0025 | -1 |
| PPAP2B | cg03136567 | chr1 | Body | 31067 | 0.05 | hypermethylated | 0.0025 | -1 |
| PRDX6 | cg03945538 | chr1 | Body | 42237 | 0.04 | hypermethylated | 0.0162 | -1 |
| SEPT2 | cg06689416 | chr2 | Body | 11073 | 0.09 | hypermethylated | 0.0040 | -1 |
| SLC1A2 | cg04451259 | chr11 | Body | 2121 | 0.09 | hypermethylated | 0.0075 | -1 |
| SLC1A3 | cg07254421 | chr5 | Body | 21690 | 0.05 | hypermethylated | 0.0450 | -1 |
| SLC1A3 | cg16963869 | chr5 | 1stExon | 45388 | 0.04 | hypermethylated | 0.0450 | -1 |
| SLC4A4 | cg11363527 | chr4 | Body | 30447 | 0.07 | hypermethylated | 0.0045 | -1 |
| SLC4A4 | cg16862063 | chr4 | 5UTR | 37613 | 0.08 | hypermethylated | 0.0045 | -1 |
| SPON1 | cg24556573 | chr11 | Body | 17860 | 0.10 | hypermethylated | 0.0302 | -1 |
| SPON1 | cg10611451 | chr11 | Body | 23372 | 0.07 | hypermethylated | 0.0302 | -1 |
| SPON1 | cg14060382 | chr11 | ExonBnd | 29879 | 0.11 | hypermethylated | 0.0302 | -1 |
| SPON1 | cg02394317 | chr11 | TSS200 | 44190 | 0.05 | hypermethylated | 0.0302 | -1 |
| SSPN | cg02058108 | chr12 | TSS200 | 19162 | 0.06 | hypermethylated | 0.0131 | -1 |
| UBE2L3 | cg13293237 | chr22 | 3UTR | 21662 | 0.05 | hypermethylated | 0.0048 | 1 |
| UBE2L3 | cg22193913 | chr22 | Body | 32873 | 0.06 | hypermethylated | 0.0048 | 1 |
| WDR82 | cg16364629 | chr3 | Body | 11772 | 0.13 | hypermethylated | 0.0073 | -1 |
| ZNF23 | cg13264964 | chr16 | TSS1500 | 24860 | 0.05 | hypermethylated | 0.0321 | -1 |

Ctrls. = Controls, p adj.P.Val. = adjusted p-value, CHR = chromosome, TSS = transcription start site, GM = grey matter, 5’UTR = 5’ untranslated region, 3’UTR = 3’ untranslated region

Statistics gene expression: Data analysis is based on a bayesian model calculation the m-value. M -1 = decreased expression, 2-fold change of mean expression. M + 1 = increased expression, 2-fold change of mean expression. P-value calculated according to the Benjamini-Hochberg correction (p<0.05).

Table 5 Overview of DNA methylation and transcriptomic studies

| Study | Analysis | Tissue | No. MSA patients | No. controls | Methods | Epigenetic modifications analysed |
| --- | --- | --- | --- | --- | --- | --- |
| de Boni  et al. | EWAS | Post mortem occipital cortex, FACS-sorted NeuN positive neuronal nuclei | 7 | 7 | EPIC array | Total methylation |
| Bettencourt et al. | EWAS | Post mortem white matter  Discovery: cerebellum, frontal cortex, occipital cortex  Follow up: cerebellum only | Discovery: 10  Follow up: 16 MSA mixed phenotype: 8 MSA-P: 8 MSA-C: 8 | Discovery: 6 Follow up: 16 | EPIC array | Total methylation |
| Rydbirk  et al. | EWAS | Post-mortem prefrontal cortex, bulk brain (grey + white matter) | 33 | 30 | EPIC array | 5-mC + 5-hmC separately |
| de Boni  et al. | Transcriptomic gene studies | Post-mortem occipital cortex | 4 | 4 | Affymetrix Human Genome U133 | - |
| Piras et al. | Transcriptomic gene studies | Post mortem white matter, cerebellum (cohort 1 and 2), post mortem LCM-dissected oligodendrocytes, cerebellum (cohort 1 LCM) | Cohort 1: 19  Cohort 2: 48  Cohort 1 LCM: 6 | Cohort 1: 19, Cohort 2: 48, Cohort 1 LCM: 6 | Illumina HiSeq 2500 | - |
| Mills et al. | Transcriptomic gene studies | Post mortem frontal cortex, white and grey matter analyzed seperately | 6 | 6 | Illumina HiSeq 1000 | - |

No. = number, EWAS = epigenome-wide association study, LCM: laser microdissected, 5-mC = 5-methylcytosine, 5-hmC = 5-hydroxymethylcytosine

Table 6 Overlapping CpGs identified in the studies of Bettencourt et al. and de Boni et al. (top 10k ranked CpGs)

|  |  |  |  | Bettencourt et al. WM | | |  | de Boni et al. GM | |  |
| --- | --- | --- | --- | --- | --- | --- | --- | --- | --- | --- |
| Gene name | CpG ID | CHR | Gene location | Delta beta | P-Value | FDR | Total Methylation MSA vs. Ctrls | Combined  Rank | Delta beta | Total Methylation MSA vs. Ctrls. |
| KBTBD11 | cg01540522 | 8 | 5’UTR | -0.02 | 1.74E-04 | 0.647 | hypomethylated | 18546 | 0.06 | hypermethylated |
| MTHFD1 | cg16041798 | 14 | Body | -0.04 | 1.10E-04 | 0.625 | hypomethylated | 14086 | 0.06 | hypermethylated |
| RPN1 | cg01163369 | 3 | TSS1500 | -0.03 | 5.86E-05 | 0.577 | hypomethylated | 42113 | 0.08 | hypermethylated |

CHR = chromosome, WM = white matter, GM = grey matter, TSS = transcription start site, 5’UTR = 5’ untranslated region,

FDR = false discovery rate

Table 7 Overlapping CpGs identified in the studies of Rydbirk et al. and de Boni et al. (top 10k ranked CpGs).

|  |  |  |  | Rydbirk et al., bulk brain | | | de Boni. et al., GM | | |  |
| --- | --- | --- | --- | --- | --- | --- | --- | --- | --- | --- |
| Gene | CpG ID | CHR | Gene location | Methylation fraction | Delta Beta | Adj.P | Methylation fraction | Combined  rank | Delta  beta | Methylation  MSA vs. Ctrls. |
| KIAA1462 | cg15274877 | 10 | Body | 5mC | -0.09 | 0.1 | Total methylation | 30389 | 0.06 | hypermethylated |
| KIAA1614 | cg11143671 | 1 | Body | 5mC | -0.12 | 1.0 | Total methylation | 14785 | 0.07 | hypermethylated |
| LINC01495 | cg02167618 | 11 | TSS200 | 5mC | 0.04 | 0.2 | Total methylation | 30430 | 0.05 | hypermethylated |
| MAPT | cg05772917 | 17 | 5_UTR | 5mC | -0.01 | 1.0 | Total methylation | 21431 | 0.06 | hypermethylated |
| MAPT | cg15926850 | 17 | Body | 5mC | 0.003 | 1.0 | Total methylation | 38909 | 0.07 | hypermethylated |
| MAPT | cg15926850 | 17 | Body | 5hmC | 0.01 | 0.9 | Total methylation | 38909 | 0.07 | hypermethylated |
| NFIA | cg14685778 | 1 | Body | 5mC | 0.05 | 0.1 | Total methylation | 21820 | 0.08 | hypermethylated |
| PARK2 | cg22222922 | 6 | Body | 5mC | 0.03 | 1.0 | Total methylation | 24258 | 0.06 | hypermethylated |
| PARK2 | cg22222922 | 6 | Body | 5hmC | -0.02 | 0.9 | Total methylation | 24258 | 0.06 | hypermethylated |
| PIM3 | cg08507178 | 22 | 3_UTR | 5mC | 0.09 | 0.2 | Total methylation | 19136 | 0.05 | hypermethylated |
| PRPF6 | cg23992672 | 20 | Body | 5mC | 0.08 | 0.2 | Total methylation | 21588 | 0.06 | hypermethylated |
| PYY | cg16789592 | 10 | TSS1500 | 5hmC | 0.06 | 1.0 | Total methylation | 28840 | 0.07 | hypermethylated |
| SLC1A4 | cg25534249 | 2 | TSS1500 | 5mC | -0.02 | 0.9 | Total methylation | 35378 | 0.05 | hypermethylated |
| SLC1A4 | cg25534249 | 2 | TSS1500 | 5hmC | 0.01 | 0.9 | Total methylation | 35378 | 0.05 | hypermethylated |
| SNCA | cg06176111 | 4 | Body | 5mC | 0.01 | 0.8 | Total methylation | 8805 | 0.07 | hypermethylated |
| SNCA | cg06176111 | 4 | Body | 5hmC | 0.02 | 1.0 | Total methylation | 8805 | 0.07 | hypermethylated |
| XRCC3 | cg09922085 | 14 | Body | 5mC | -0.15 | 1.0 | Total methylation | 47948 | 0.08 | hypermethylated |

CHR = chromosome, WM = white matter, GM = grey matter, 5-mC = 5-methylcytosine, 5-hmC = 5-hydroxymethylcytosine

Table 8 Overlapping DEGs identified in the studies of de Boni et al. and Mills et al.

|  |  |  | de Boni et al.  Affymetrix gene expression analysis, occipital cortex, significantly changed grey matter DEGs, MSA vs. Ctrls. | | Mills et al.  Illumina HiSeq 1000, bulk brain tissue, frontal cortex, grey matter DEGs significantly upregulated compared to WM in MSA patients | |
| --- | --- | --- | --- | --- | --- | --- |
| Gene | Gene name | CHR | M-value | P adj. p-value | Fold change | q-value |
| ABHD8 | abhydrolase domain containing 8 | 19 | 1 | 0.03 | 7.0 | 0.001 |
| CAMKK2 | calcium/calmodulin-dependent protein kinase kinase 2, beta | 12 | -1 | 0.003 | 6.1 | 0.002 |
| COL5A2 | collagen, type V, alpha 2 | 2 | -1 | < 0.001 | 18.6 | 0.0001 |
| CXCL14 | chemokine (C-X-C motif) ligand 14 | 5 | -1 | 0.002 | 12.6 | 8.13E-06 |
| LY6H | lymphocyte antigen 6 complex, locus H | 8 | 1 | 0.002 | 4.3 | 0.01 |
| SLC4A4 | solute carrier family 4, sodium bicarbonate cotransporter, member 4 | 4 | -1 | 0.005 | 4.9 | 0.004 |
| WIF1 | WNT inhibitory factor 1 | 12 | -1 | 0.01 | 15.2 | 0.01 |

Data analysis is based on a bayesian model calculation the m-value. M + 1 = increased expression, 2-fold change of mean expression.
